# The London Lancet for April, on Fractures, Hemorrhage, and Gangrenous Ulcers

**Published:** 1850-06

**Authors:** Theodore S. Bell

**Affiliations:** Louisville, Ky.


ART. IV.—*The London Lancet for April, on Fractures, Hemorrhage, and Gangrenous Ulcers.* By THEODORE S. BELL, M. D., Louisville, Ky.

In the February number of this Journal, an attempt was made to institute a comparison between the results of the treatment of serious injuries, as displayed in the best hospitals of Europe, and the results of the bandage treatment in the same kind of injuries. It is very clear, I think, that all valid medical logic, all the pathological characters of that class of diseased or deranged action, for which the bandage was commended in the essay alluded to, and the vast benefits found exclusively in the principles of the bandage, restricted in their proper place, point emphatically and impressively to the necessity of urging the attention of medical men, to correct conceptions of the value of the roller as a surgical agent. I have been forcibly reminded of this necessity in reading the April number of the London Lancet.

In that valuable journal, the following cases are detailed: "Ligature of the Femoral Artery, for a Wound of the Anterior Tibial, followed by Amputation;" "Compound Oblique Fracture of the Tibia, with Comminuted Fracture of Fibula;" "Compound Comminuted Fracture of Tibia and Fibula, in the London Hospital;" "Gangrenous ulceration of the hand." The lessons derived from success are not always the most forcible and impressive; those that spring from fatal issues enforce themselves vividly on the mind, sometimes, and guide to better methods of treatment. Experience, observation and reading constantly urge the utility of the bandage, in a vast range of maladies and injuries, and I should hold myself recreant to science, in neglecting any useful opportunity for presenting this important subject to medical readers.

Impressed with these views, the reader of these remarks may find much valuable instruction in the following cases:

W. S. Davis, a Member of the Royal College of Surgeons, reports that a robust, temperate farm-laborer, æt. twenty-eight, accidentally wounded himself in the anterior part of his leg, with a reaping hook. The surgeon found the patient, about two hours after the accident, very faint from loss of blood, although the hemorrhage had been effectually arrested by a handkerchief, bound tight around the leg. This dressing was not removed by the surgeon, but in addition to the handkerchief, a tourniquet was loosely applied over the upper third of the femoral artery, with directions to have it tightened in case of a return of bleeding. In four hours after this, the patient had rallied, and there had been no return of hemorrhage. The symptoms continued in this favorable condition up to the sixth day after the wound, when the hemorrhage returned, and nearly prostrated the patient before his wife could arrest the bleeding with the tourniquet. The femoral artery was tied, and for two days the patient seemed to do well. On the third day from the operation, hemorrhage occurred again; on the fifth day the hemorrhage returned, and on the sixth day, being the twelfth from the time of the accident, this unfortunate patient was subjected to the loss of his leg. His surgeon was not at all satisfied that the patient had received the best treatment. In the report of the case, we find the opinions of several hospital surgeons appealed to, one of whom says: "I am not prepared to say the case could have been better managed. In nine cases out of ten, your first operation of ligaturing the superficial femoral artery would have sufficed to control all hemorrhage, but the tenth case (such I consider yours) has induced surgeons of authority to lay down the rule, that an artery, when wounded, should be cut down upon, and both ends secured; of course there are exceptions to every general rule, and your case might fairly be considered one."

Now I appeal to the readers of this Journal, to say whether it is at all probable that this patient enjoyed the resources of surgery in the treatment of his case? Was there the slightest necessity for a return of the hemorrhage, after its primal arrest? Is there any reason for tying up the femoral artery, for a wound of the anterior tibial? And finally, is it not humiliating to think that such a hemorrhage as this was so managed as to result in the loss of a leg?

These are the considerations that make this case so interesting in reference to the treatment of hemorrhages by the roller. Of the power of the compress and *partial* bandage, we have ample proof in this case. If the surgeon had applied a roller properly to this limb, at his first visit, after placing compresses in their proper places, there would have been no kind of trouble subsequently. The hemorrhage was perfectly controlled for some days, with no better dressing than a common handkerchief, which acted as a ligature. If the whole limb had been enveloped in a roller, greater compression could have been made, if necessary, and the surgeon would have saved his time, his feelings, his arguments and appeals to other surgeons, and the poor laborer would have saved his limb. It is shameful that a temperate, robust farm laborer, aged twenty-eight years, should have received the treatment this man did from a member of the Royal College of Surgeons. There could have been no kind of difficulty in controlling the hemorrhage in this case, because we have proof of the fact in the details furnished, and we are constrained to say that the patient was a victim to a very bungling and outrageous surgery. Reader, be guarded, and do not permit such offensive practices to creep in upon your surgical management.

The next case, that deserves attention is one of "compound oblique fracture of the tibia, with "comminuted fracture of the fibula." This complication occurred in a man aged fifty-two. He fell into a saw-pit, and produced the injury. A piece of bone, one inch and a half, protruded through the stocking, and there was profuse hemorrhage, from a slight laceration of the anterior tibial artery. The tibia was broken downwards and inwards immediately above the inner malleolus. The fibula was broken above the outer malleolus, and again about two inches higher up. This serious injury was complicated with dissipated habits, and an ulcerated condition of the leg. The surgeon determined, very properly to give the patient the benefit of an attempt at restoration, without amputation. The means resorted to were *debridement*, a compress over the wounded artery, the use of Liston's splint, and the double inclined plane. This was continued four days, and on the removal of the applications, it was discovered that *the bone was protruding as badly as ever*. The reason given for this is: "*That the fracture being below the attachment of the soleus altogether, that muscle acting from the os calcis as a fixed point, pulled the superior fragment downwards and inwards.*" The reduction of the broken bone to its proper apposition, and the maintainance of the fragments in that condition, were the indications to be fulfilled in this case, and the means for obtaining these results are obvious. The pullies would have accomplished the reduction, and the roller would have perfectly controlled the soleus muscles. There is nothing in mathematics more certain than both of these facts. Yet the patient, in the case before us, had to submit to muscular spasms, which dragged the bone from its place, and extensive suppuration took place, in the vicinity of the ancle-joint. Upwards of a month after the accident, half an inch of the protruded bone was removed with cutting pliers, and nearly two months after the accident, the surgeon congratulated himself that he had at length succeeded in applying an apparatus which effectually controlled the muscle, and kept the limb in position! But under proper treatment this patient should have been walking about, at a time, when his surgeon had just discovered a method of controlling muscular action! This discovery was a combination of Liston's and Boyer's splints!

Dr. Carter, the surgeon in the case alluded to, says: "If you can only procure an apparatus which shall resist muscular action completely, you need not attend to position (*quoad* muscular action) at all; it becomes necessary only to relax the fibre when one cannot tire it out by perfect resistance." The bandage has a greater capacity for "resisting muscular action completely," than any other agent in the hands of the surgeon, but no use was made of its powers in this case, beyond binding a splint on the limb. If the limb had been properly treated, we repeat, and we speak, not only from inductive reasoning, but from personal observation, this patient would have been saved from a great deal of suffering, and would have been walking about much sooner, than he is said by his surgeon to have been hobbling about on crutches. And the surgeon would, with the roller treatment, have turned out a limb, that would have been much better than "a wooden one." The patient seemed to think that his shortened limb, which made him a cripple for life, was so useful that "he would not exchange it for a wooden one for all Europe."

The above is an instructive case, and is well worth the study of the practitioner of medicine. From the pit-fall of bad surgery, manifest in this case, a great deal of useful instruction springs to our view, and urges us to avoid similar treatment, if we would avoid analogous evils. There is not a muscle connected with fractures in the human system, that cannot be effectively controlled by the roller, and the reader cannot fail to see, that all the serious defects of the treatment of the case, we have detailed from the April number of the *London Lancet*, arose from the inability of the surgeon to control muscular action. If the reader remembers the quotations made from Maclise, in the article, on the bandage, published in the February number of this *Journal*, and the remarks that accompanied Maclise's anatomical statements, he will feel forcibly impressed by Dr. Carter's case. The use of "splints" is a great hindrance to the progress of surgery, and an evil to patients, as a general rule. In a long experience in fractures, I have yet to see one that did not yield readily and properly to the bandage, with but little trouble to the surgeon, and great diminution of the suffering of the patients, compared with cases treated in a different manner.

Another case somewhat resembling the one already detailed, is mentioned in the London Lancet for April. The case was treated in the London hospital. On the 30th of September, a boy, eleven years old, was admitted into the hospital, with a compound fracture of tibia and fibula, caused by his jumping out of a window, fifteen feet above ground. "There was a wound about three inches above the ancle joint, directly across the tibia, through which the fractured bone was protruding for about a quarter of an inch." The fracture involved the fibula, and "was of a very comminuted nature." There was a great deal of hemorrhage. Pressure was applied, *the limb settled on a side splint*, and a dose of opium administered. *Reaction was very considerable*, and towards the middle of October, three large abscesses formed along the course of the tibia. They were opened, and discharged a large quantity of pus. *The child's health now began to suffer;* he had quick pulse, flushed countenance, brown tongue, &c. The propriety of amputation was a matter of debate, but the healthy aspect of the wound, and the extension of the abscesses being such as to demand the amputation of the thigh, plead for delay. Stimulants, tonics, full diet, and *pressure upon the abscesses* were resorted to, and the limb was slung in the hospital apparatus. The sinuses healed, the boy's health improved, and, according to the report, is likely to do well.

In the foregoing case there is a repetition of those unnecessary evils that so constantly meet the reader in reports of European surgery. The youthfulness of this boy, the fracture near the joint, the hemorrhage, the "considerable reaction" which occurred immediately after the injury, all pointed to the necessity of the roller treatment.

That would have controlled the hemorrhage, and the re-action, and prevented all the constitutional suffering, connected with the formation of abscesses. There is one point in cases of fracture that cannot be too indelibly engraven on the mind of the practitioner, and that is, that his patient is always in danger, from the moment that he permits suppuration to take place in a fracture contiguous to a joint. There is no reason why pus should be permitted to form in any case of the kind, and it is as much the duty of the surgeon to guard against such a result, as it is to see that ends of fractured bones are in apposition, or to maintain them in that condition. The reasons for the danger of purulent collections, will be shown in a subsequent part of this paper.

The following is a case that is filled with impressive admonitions on the perils of suppurative action in a wound. The particulars of the case are gathered from the London Lancet for April.

A patient, aged forty-five, was admitted into St. Thomas's hospital on the 18th August, under the care of Mr. Solly, with a compound fracture of the fourth and fifth metacarpal bones of the left hand, with much laceration of the soft parts. The wound was received from a circular saw; the patient had always enjoyed good health, but was intemperate in his habits.

The hand and forearm were placed on a flat splint, and cold water dressing applied. "There was no sloughing, and he soon recovered from the effects of the accident." Up to the 16th September, the case is reported to have done remarkably well, not having shown a bad symptom. "The wound was healing fast; a healthy granulating sore about an inch long, and half an inch broad, remaining open." But stress is laid upon the fact, that on the 16th of September, a new nurse, who had just entered upon her duties at St. Thomas's, dressed the wound, "by applying straps of plaster very tightly over the whole sore."

There is an attempt to ascribe the untoward symptoms that supervened, to this interference of the nurse, but it is absurd to attempt such a thing.

The report goes on with a detail of the symptoms, from day to day. On the 19th of September the sore began to spread, and instead of adhesive strips and the bandage, the surgeon ordered a linseed meal poultice. On the day after, it appeared that the sore had spread much during the night, and commenced sloughing, attended with much pain, and some slight shivering. During the day the sore spread rapidly under the combined influences of ulceration and sloughing. "The hand swelled, and around the edges of the sore, for some distance, there was a dusky red inflammation of the skin. Much aching pain, the skin is hot, pulse one hundred and ten, full, but very compressible; face flushed; tongue dry and brown; appetite bad; head-ache, and great thirst." Here is a frightful array of symptoms, showing itself the day after pressure was removed from the sore, and a linseed meal poultice substituted. On the 16th, a slight change in the condition of the ulcer was attributed to the use of adhesive strips, but the surgeon does not seem to regard the great change he made in the treatment, on the 19th, as connected with the untoward symtoms that were coincident with the change of treatment. Nor do I regard these untoward symptoms as the result altogether of that change, but there is more reason for saying they were, than there was for ascribing "the angry appearance of the sore," on the 17th, to the use of adhesive plaster straps, on the 16th. If the hand had been dressed properly with strips, and a roller, and constitutional treatment had been commenced, on the 16th, this patient would have been saved. The angry appearance of the sore on the 16th, and the symptoms up to the 20th, showed that "diffused inflammation" had extended to the thoracic and abdominal viscera, a species of danger that should never be lost sight of.

On the 21st, nearly all the symptoms were aggravated. The stale beer grounds poultice, and nitric acid applied to the sore, constituted the local prescription. The constitutional treatment was feeble almost to the verge of homœopathy.

On the 22d, 23d, 24th, indeed up to the 3d October, the case seemed to make no improvement. The constitutional symptoms varied somewhat, but the sore continued its sloughy condition. On the 4th the slough separated, and healthy suppuration was established. On the 11th the case commenced retrograding, on the 12th commenced sinking, *and, under an aggravation of the constitutional symptoms*, the patient died on the 14th. The *post mortem* revealed invaluable lessons. The wrist joint contained thick, turbid pus. The radial artery contained many patches of atheromatous, and a few of bony matter; its inner surface was rough, and the ulnar was in much the same condition. The brachial was slightly diseased. The basilic vein was filled at its upper part with thick, white, healthy pus. All the veins leading from the ulcer were swollen, and filled with pus, their coats being thickened. In the anterior part of the left lung, near its base, was an oval mass of purulent infiltration in its early stage; the air cells were filled with pus. The abdominal viscera generally healthy; there was recent lymph on portions of the peritoneum, and there was perforation of the intestines, in the terminal portion of the transverse colon, the mucous membrane of which was studded with ulcerations, in irregular patches.

Samuel Solly, Esq., M. R. C. S., made some clinical remarks on this case, that bring up the matter, which I wish to impress upon the reader's memory. Mr. Solly said: "*The veins leading from the wound were charged with pus. These vessels absorbed the pus, carried it into the*

*system, and poisoned him!*" This is certainly a more philosophical cause for the angry condition of the ulcer, on the 16th of September, than the one to which that condition was ascribed. The possibility of such results, as are found in the case under review, should admonish the surgeon against neglecting the only agent that can exercise any control over purulent secretion. If the hand had been dressed, originally, with the roller, and constitutional treatment had been resorted to, the wound could not have assumed an angry appearance; and even after the retrograde tendency manifested itself on the 16th September, the roller treatment would have prevented that dreadful state of things, that continued with varying results, up to the 14th of October. If the roller had nothing to commend it to the attention of medical men, beyond its power over purulent formations after fractures, injuries of the joints, gun-shot wounds, &c., it would still be the greatest blessing in the hands of the surgeon. It will not be out of place now to take a view of some of the phenomena of purulent inflammation. Of all the matters presented to the consideration of the surgeon, in treating serious injuries that are curable, the proper management of the case so as to prevent purulent secretion, or to control its movements, after it has taken place, is one of the most important. It is in this field of curative power, that the roller successfully vindicates its claim to the attention of the philosophic surgeon, and clearly shows how much nature may be indebted to art in the removal of evils in the animal economy. The great range of the usefulness of the bandage has been shown in my former essays on the subject, but its salutary, beneficent agencies in the treatment of purulent secretion have not been dwelt upon sufficiently.

As a proper preliminary view of the pathological phenomena, and effects of purulent secretion, it will not be considered amiss, if the reader's attention is called to the microscopic revelations of the phenomena of purulent formation. Copland gives the following graphic account of these:

"The formation of pus in an inflamed surface or tissue takes place as follows, according to the observations of Kaltenbrunner, Gendrin, Carswell, and the author. In the field of a microscope the inflamed capillaries seem uniformly red, and the circulation in them is retarded or has ceased. Serum and coagulable lymph are effused in the areole of the tissue; and, if the inflammation is very intense, the exuded fluid is more or less colored by the exudation of red globules or of blood. The whole of the inflamed part is quite opaque. As soon as suppuration commences, the red color begins to disappear in various points, giving place to a yellowish granular-like matter in the capillaries, and connecting cellular tissue. In the centre of the inflamed tissue, several of the capillary vessels, which were obscured by the accumulated blood, reappear, some containing red, others yellowish-gray globules, which gradually become more distinct, increase in number and size, begin to move slowly, and, traversing the capillaries, arrive at the surface of the tissue, or at the edges of the solution of continuity, if this has occurred, in the form of globules of pus (Carswell.) Gendrin states that he has distinctly seen the globules of blood, after stagnating in the capillaries of the inflamed part, losing their coloring envelopes, becoming opaque, and assuming a grayish yellow color, approaching to that of pus; and that he has traced them moving slowly in the capillaries, and, as they advanced to the suppurating surface, gradually acquiring all the characters of pus. The observations of Kaltenbrunner agree with those of Gendrin as to the transformation of the blood-globules into the globules of pus, and as to this taking place *within* the capillaries; but they also seem to prove, what I have observed in several instances, that the red globules, or blood, exuded in an intense state of inflammation into the areole of the tissue, undergo a similar change to that which takes place within the capillaries when the circulation becomes stagnant in them; and that pus may thus be formed *without*, as well as *within*, the capillaries of an inflamed part, the fluid portion of the secretion consisting of the serum of the blood. Kaltenbrunner even supposes that not only the blood of the inflamed tissue, but likewise a part of the tissue itself is converted into pus-globules."

"From these facts it is evident that, in an inflamed part, certain changes precede the formation of pus: 1st. A loss of the vital tone, or a change of vital action in the extreme capillaries. 2d. A retardation or stagnation of the circulation, and partial coagulation of the blood in them. 3d. A change of the blood-globules into pus-globules, and the discharge of the latter with a portion of serum on the suppurating surface. 4th. A similar change of the globules of blood extravasated in the inflamed part, these globules losing their coloring envelopes, and becoming the globules of pus."

There is scarcely a day of a surgeon's life, when it is not essential that these principles and facts shall be present to his mind. They are guides that lead him through intricate labyrinths; they are lights that enable him to "see by faith, and not by sight," and reveal vital knowledge to him, as perfectly as though it were an object of one of the senses.

In connection with these facts the following admirable remarks, on the changes observed in the blood in sthenic inflammation, are quoted from the British and Foreign Medical Review:

"I. *a.* The quantity of *fibrin* in the BLOOD undergoes a decided increase; the *plasticity* of the whole mass, therefore, but especially that of the liquor sanguinis, is greatly augmented. *b.* There is a corresponding increase in the proportion of *white* corpuscles, which are present in large amount in the vessels of the inflamed tissues, and have a great disposition to adhere to their walls, but which are also present, to an unusual amount, in the entire mass of the circulating blood. *c.* The increase in the proportion of fibrin is chiefly a local action, exerted on the blood during its passage through an inflamed part, and probably effected by the instrumentality of the *white* corpuscles. *d.* There is usually an increase, not only in the *quantity* of fibrin, but in its *plasticity* or tendency to become organized, as shown by the greater perfection of the fibrous structure into which it passes in coagulating. This may consist in an increased attraction between its particles, which continues to operate for some time, causing contraction of the fibrous net-work, subsequently to its first production. *e.* There is also an increased attraction between the red particles of blood, causing them to adhere together in rolls more firmly and for a longer period than they do in healthy blood. *f.* To these two causes, usually aided in their operation by the slowness of the coagulation, all concurring to produce an increased tendency to separation between the red corpuscles and the liquor sanguinis, we may ascribe the production of the buffy coat of inflammatory blood. *g.* The increased *plasticity* of the blood is so constant a phenomenon of inflammation, that it may be regarded as essential to the presence of that state.

"II. *a.* On the other hand, the *formative power* of the inflamed TISSUES appears to be diminished; their usual functions, whether of nutrition or secretion, being completely checked, or insufficiently performed, or perverted in their character. *b.* While, therefore, an over-production of fibrin is taking place in the blood, there is diminished consumption or appropriation of it in the tissues. *c.* If the inflammation be severe in its character, the vitality of the tissues is so diminished as to cause, not only a cessation of their formative actions, but also an increased tendency to disintegration, as shown in *suppuration* and *ulceration;* or positive death of a large part, as in *gangrene.* *d.* The depression of the vitality of the tissues sometimes appears to result from a previous over-excitement of it, as when inflammation follows excessive use of a part, or the application of stimulants to it; but it is sometimes the consequence of some directly sedative action, as that of cold. *e.* Hence both *determination* of blood and *congestion* have a tendency to produce inflammation; the one being a state of over-excitement, which is very prone to occasion subsequent depression, while there is at the same time a tendency to increased production of fibrin in the blood, the other being itself a state of depression of formative power in the solids, but not passing into inflammation, unless there be at the same time an increased plasticity of the blood.

"III. *a.* The MOTION OF THE BLOOD in the capillaries of the inflamed part is greatly retarded, as we might have anticipated from the impairment of the functional operations of the solids. There may even be a total stagnation of the blood in the capillaries of a considerable portion of the tissue, which will be followed by its death and disintegration. The degree of stagnation will depend upon the amount of the depression of the vitality of the surrounding parts. *b.* The motion of blood through the vessels in the neighborhood, however, is more rapid than usual, and these vessels are themselves enlarged; so that the total quantity which passes through an inflamed member in a given time is greater than usual. *c.* The vessels are enlarged both *in* and *around* the inflamed part, in consequence of a diminution of the tonic contractility of their walls, which causes them to admit of abnormal distention by the impulse which the blood receives from the heart. This diminution is another evidence of the depression of the vital properties of the solid tissues in an inflamed part.

"IV. *a.* The PRODUCTS of inflammation differ from those of the ordinary processes of nutrition and secretion, not so much in their materials as in the nature of the change which these have undergone. *b.* When the intensity of the inflammatory process is moderate, the *liquor sanguinis,* containing an unusual proportion of fibrin, and possessing a high degree of plasticity, is effused into the neighboring tissues or upon the neighboring surfaces, being generated, by the local actions of the part, faster than it can be withdrawn by its formative processes. By the organization of which it is susceptible, when in contact with the living solids, it spontaneously assumes the form of simple fibrous tissue, constituting false membranes on the surface, or consolidating the substance into which it is effused. *c.* If the inflammatory process goes no farther there is no disintegration of the original tissue; but if its vitality be too far depressed it dies; and the changes which it consequently undergoes impress themselves upon the fibrinous effusion. The fibrin loses its vital power of coagulation, and in this aplastic state becomes the chief ingredient in the *liquor puris;* while the cells *(pus-corpuscles,)* which are found floating in it, resemble the white corpuscles of the blood in a degenerated form. *d.* When the inflammation is very severe, and the stagnation of blood in the capillaries of the part is complete, an entire loss of vitality in the whole tissue at once, or *gangrene,* is the result. Gangrene does not originate, however, in inflammation alone, since any other cause, such as the long-continued action of cold or pressure, interrupting the capillary circulation, or obstruction to the supply of blood through the arterial trunks, will equally produce it, by the suspension of the formative processes thus occasioned. But unless some degree of inflammatory action, that is, an increase in the plasticity of the blood, be set up at the same time, there is an indisposition to the formation of the line of demarcation between the sound and the dying parts, and the gangrene has a tendency to spread."

The subject is now fairly before the reader, and he can have no kind of difficulty in seeing why the bandage has all those important and invaluable uses, ascribed to it by all surgeons who have enjoyed an experimental and observational acquaintance with it. It is difficult to imagine how anything else in the treatment of the most usual fractures, the generality of ulcers, and a variety of gun-shot wounds, was ever thought of. What kind of comparison can be instituted between the treatment of fractures by splints, and by the roller; of ulcers, by hundreds unguents, and by the adhesive strips and the bandage, and of gun-shot wounds, by those means that invite gangrene, and the roller, which prevents it? Who can look upon the phenomena of sthenic inflammation, so graphically and clearly described, in the above quotation from the British and Foreign Medical Review, and imagine any surgical agent that boasts a thousandth part of the beneficent uses of the roller?

But there are other important considerations that belong to this subject of purulent secretion. From the facts contained in the quotations above, it is clearly seen how beneficial the bandage may be in its control over the local phenomena of the formation of pus, but this is of small moment compared with the constitutional effects. These effects are very conclusively shown, in an essay on "Secondary Abscesses," published by Dr. John Watson, in the American Journal Medical Sciences, and quoted by Dr. Lee, in the American edition of Copland's Dictionary of Practical Medicine. The reader will necessarily have to travel over some of the ground he has already been over, but he had better do that once too often, than not often enough by one time. Dr. Lee thus quotes Watson:

"By a variety of cases, whose history is given, Dr. W. shows: 1st. That injuries and surgical operations not dangerous in themselves, may be followed by severe, and even fatal, secondary affections in distant parts of the body, and in organs essential to life; and that the pathological states, thus induced, are not necessarily connected with a previous external injury, inasmuch as they may be induced, independent of such injury, by disease arising spontaneously in the system. 2d. That the most usual pathological appearances found after death in these cases are: depositions of purulent matter, inflammatory congestions, effusions of coagulable lymph, sero-purulent effusions, effusions of sanies or bloody serum, adhesions of contiguous surfaces, ulcerations, and disorganizations of different structures, as of the eye and of the tissues about the joints. The whole of these pathological conditions are not often found existing together; many of them, however, may occur in the same case; the tissues within which these morbid changes are most common are the cellular tissues, whether subcutaneous, parenchymatous, or intermuscular; the serous tissues, as of the thorax, the abdomen, and encephalon; the synovial membranes, the skin, the muscular texture, &c. The organs most frequently affected are the lungs, the liver, the brain, the spleen, and the knee and shoulder joints, but almost any part of the body may become the seat of these secondary disorders. 3d. In all cases of the kind under consideration, where thorough examination was made, the inner coats of the veins in the neighborhood of the primary injury, and in other parts of the system, were found inflamed. In some cases the arteries also were found diseased; but inflammation of the inner coats of arteries, unlike that of veins, appears to have no necessary connection with the other consecutive affusions. 4th. That in cases of secondary abscess, and other consecutive disease connected with inflammation of the veins, purulent matter was generally, but not in all instances, found within the cavity of the veins. It is not yet fully established whether phlebitis is always attended with secondary local affections; though there is considerable reason to believe that such is the fact. 5th. That the purulent matter formed within the veins may be either blocked up by a barrier of lymph, or exist in a free sta'e, without any such barrier to prevent it from entering into the circulation, and contaminating the blood. But the secondary abscesses are known to occur in connection with phlebitis, whether the pus in the veins be free or circumscribed; and even when no purulent matter has been detected, either in the orig nal wound itself, or within the veins leading from it. 6th. That purulent matter existing free within the cavity of the veins does not necessarily lead to the formation of secondary abscesses in the viscera. 7th. That besides these secondary disorders connected with phlebitis, there are others occasionally resulting indirectly after operations, &c., of an entirely different description, and so far as we are able to judge, in no way connected with disease of the veins. Such, for example, as appear during the existence of high inflammatory excitement, or come on more slowly after the system has been much exhausted by contracted disease and hectic fever.

Dr. W. maintains that secondary depositions, and other pathalogical phenomena connected with them, are not, in all cases, if indeed in any, simply the result of absorption, and subsequent transposition of pus from a remote part of the system; and that as they occur in all parts of the body, without any definite relation to the part primarily affected, they cannot be attributed to any special sympathies between the parts first diseased and those subsequently affected; rejecting also the doctrine which imputes local violence to the part secondarily diseased; as well as that of constitutional irritation, which, besides its indefiniteness, throws no light on the manner in which the constitution becomes involved. Dr. W. believes that the doctrine which ascribes secondary abscesses to a vitiated condition of the blood, induced by the purulent matter, or other morbid exhalations of inflamed veins mixing with the blood, and thus exciting local inflammation in the parts secondarily diseased, is the one most conformable to facts, while, at the same time he admits that matter, formed originally within the veins, may subsequently be thrown out of the circulation as any other foreign or useless substance, without exciting local inflammation in the parts upon which it is deposited. The existence of pus in any part, is no proof of the previous existence of inflammation in that part."

The reader will not find his labor in vain, if he rises from the perusal of these valuable facts, with clear views of their great, their vital importance. And when he becomes an expert in the philosophical and scientific use of the roller, and sees the manifold benefits of its application, and feels by comparison, how much of suffering, and how many perils, and other evils, he has saved his patient from, he would not exchange that simple agent, for all other appliances known to surgery, in these cases.

But let us resume an examination of some of the details of the phenomena of purulent secretion. The case in St. Thomas's hospital, of which an abstract has been given in this paper, is a most valuable one in connection with all the facts and observations brought forward here, on the subject of purulent secretion. How impressively does it corroborate the conclusions of Dr. Watson! With a hop and skip philosophy that has loaded practical medicine with mountains of error, the surgeon of St. Thomas's took it for granted that the coincidence of two circumstances, stood in the relation of cause and effect! He assumed, that because the patient got worse on the 17th, the change was the result of strapping the hand, instead of using a poultice! That was a fatal piece of false logic, and led to all the subsequent disasters of the case. I think this will be manifest to any one, who has read the report of the case of carbuncle contained in the May number of this work. In that report, it is shown that a violent form of gangrene, accompanied with grave constitutional symptoms, came on while the linseed meal poultice was on the hand, and that a change of local treatment, consisting of a tight bandage to each finger and the thumb, and a roller to the hand and forearm, immediately stopped the gangrene, and relieved the constitutional disturbance. And I am as perfectly persuaded that a kindred result would have followed the same treatment, in the case in St. Thomas's hospital, as I am of any truth in therapeutics. There is not a solitary fact, connected with the subject of inflammation and purulent secretion, that does not indubitably show this to my mind, and all my experience and observation corroborate the view.

Upon these points it may be useful to detail a case which passed under my observation recently, and which was watched with considerable anxiety. A young man, for whom I entertained a warm friendship, was attacked some months since with a swelling in the inguinal glands, which terminated in a copious purulent secretion. The case was not venereal. From various causes, such as business that required him to travel, my patient's case was protracted until three large sinuses presented themselves, in the abdominal parietes. Two of these sinuses were nearly perpendicular, the other was transverse, occupying about five inches in length. They resisted all the usual means of relief, and became so threatening, that I had to confine the young man to bed. A roller was placed on the foot, and passed up the leg, to the vicinity of the sinuses. Adhesive straps were drawn lightly across the sinuses, and thick compresses laid over them, so as to command them from the bottom, to their open end. Over these the roller was drawn tightly. Purgative medicines were ordered, consisting of blue mass and ext. colocynth, in sufficient quantities to purge freely three or four times a day. From the beginning of this course, the improvement of the patient was rapid beyond my most sanguine expectations. The sinuses rapidly healed, and in two weeks, my patient was discharged; the sinuses were, at that time, so nearly well as to need no further attention, and the constitutional health was better than I had ever known the young man to possess before. This case is selected from a great number of a similar character, but the only reason for selecting this, is because it is the most recent one I have seen.

I have dwelt upon the subjects embraced in these remarks, because of their vast importance. They address themselves to the surgeon in his daily walks, and are intimately connected with true surgical art, and the interests of humanity. A man may be called upon to treat one hundred cases of the kind referred to in this paper, where he will be summoned to one case of lithotomy, or of hernia, requiring an operation. And in no other department of surgical treatment, than that connected with the common occurrences of professional life, is it more necessary to have correct views, and the use of proper remedial means. A surgeon who should apply a poultice to the perineum, for a calculous disease, or an unguent in cases of strangulated hernia, might safely be considered a little behind the times, but to my mind, the use of splints alone for fractures, and unguents and balsams for ulcers and gun-shot wounds, is quite as preposterous and futile.

But the reader must be guarded against the impression that the bandage is the sole means of treatment, in the various injuries and disorders for which it has been commended in this essay, and the preceding papers on the bandage. Constitutional treatment and a prudent regimen are not only useful in almost all cases, but absolutely essential in many. In relation to purulent secretions, Copland says: "Experience having shown the advantages of removing pus by exciting absorption, the means most effectual for attaining that end, must be pursued." The means usually resorted to, are iodine, sulphureous *douches*, frictions with mercurial, camphorated, and terebinthinated liniments, and blisters, and they are, to a great extent, useless. Among the effectual measures, for exciting the absorption of pus at the least expense to the constitution, when it can be applied, the roller is first. It controls the action of the veins, in the sthenic varieties of inflammation, and sustains them above the point of debility in the asthenic form. The next measure of importance is the use of drastic purgatives, and in an inferior degree, dieuretics and diaphoretics are useful for promoting the action of the absorbents. If these measures of control are neglected, absorption may go on at a ruinous rate. And it must be borne in mind, that the means commended here, must be persevered in, as long as heat, pain, redness, and swelling are connected with purulent accumulations.

In former essays the utility of the roller has been vindicated by its own results; in this one, its uses and benefits have been sustained by the disasters that attended its neglect. I pray the reader to ponder well upon these principles and facts recorded in the essays on the bandage, in this Journal. They are vitally concerned in all the walks of surgery; but, above all other considerations, they are most intimately connected with the highest interests of this life. They are associated with questions of deformity and symmetry, with the integrity of the body, with a crippled gait for life, and with life and death. Reader, look well into this matter, for the honor of your profession is often staked upon issues involved in the principles and facts presented to you in these quotations; the limbs or lives of your patients may hang trembling in the scale of your judgment, and upon your acquaintance with these things, and a decision founded upon accurate knowledge, may depend the weal or woe of numbers of your fellow beings. Scrutinise the facts detailed in the quotations from Dr. Watson, and the British and Foreign Medical Review, and ask yourself what evil influence is pourtrayed there, that skillful and scientific knowledge of the roller will not amend? What vital property is there in these reparative processes that will not derive material support from the bandage? Can it be a matter of surprise to any one, who looks well into these things, that in regions of surgery, where the use of the bandage is neglected, we constantly hear of extensive purulent secretions in cases that know nothing of such disasters, where the bandage is judiciously applied? Can we wonder that limbs are sacrificed on the most frivolous pretences, and lives endangered or lost, that should know nothing of peril, if surgical art were in accordance with the truths of pathological anatomy? To guard the readers of this *Journal* against the whole machinery of error in all cases of which the roller is cognizant, has been the aim of the various essays and reviews on the roller, that have appeared in these pages. And the writer of these remarks, has received ample evidence, that these labors have not been in vain.

### MISCELLANEOUS MATTERS OF PRACTICAL MEDICINE.

The number of the London Loncet from which the foregoing surgical cases were drawn, also contains a number of papers of practical value, an abstract of which cannot fail to be useful to the readers of this Journal.

*Nitrate of silver, for laceraeed wounds of the face, &c.*—

John Higginbottom, F. R. C. S., reports the great success of the prompt application of the nitrate of silver, to lacerated wounds of the face. The advantages ascribed to it are, first—the prevention of the irritation arising from the irregular edges of lacerated wounds, and the induction of adhesive inflammation, as readily in such wounds, as in those that are incised—second, the inflammation, swelling and irritative fever, consequent on lacerated wounds, are in a great measure prevented, and as there is neither ulceration, nor loss of substance, unsightly scars and raised cicatrices are avoided.

Mr. Higginbottom reports two interesting cases of wounds of the face, and one of lacerated perinæum, in which the nitrate was used with advantage. In the case of the lacerated perinæum, the edges were drawn together by the interrupted suture in two places, and the nitrate of silver applied along the skin on each side, close to the line of the wound, and on the line of the wound. On the third day the sutures were removed, and the ligature marks touched with the nitrate to prevent ulceration. "The wound united by the first intention; there was no swelling; and it is worthy of observation, that the eschar surrounding the laceration made by the nitrate of silver had the power of fixing the parts as if adhesive plaster had been applied." The catheter was not used to draw off the urine, but it should not be neglected in such cases.

The plan of treatment thus urged by Mr. Higginbottom, may be useful in wounds of the localities named, but in parts accessible to the roller treatment, the latter should have the preference.

*On beds for the sick.*—This is a subject that well deserves the attention of medical men. My attention was forcibly directed to the matter some years ago, by the perusal of the case of Dr. Nichols, of Danvers, Mass. He had been afflicted several years with a severe neuralgic affection, for the relief of which all medication, and all other means were ineffectual. He had been forced to cease all efforts at locomotion, and had resigned himself to a life of hopeless misery. In the midst of his despair, he read, or heard of Dr. Arnott's Hydrostatic bed, and had one made for himself. The use of this promptly brought relief, and restored him so perfectly to health, that Dr. N. walked several miles, to a meeting of the Massachusetts medical society to deliver the lecture on his case, which I read with pleasure and profit. The hydrostatic bed is invaluable in many cases. Some years ago, I was called to a gentleman from Mississippi, who had an acuteness of neuralgic suffering far beyond anything I have ever heard of in any other case. For three years he had never dared to let a ray of light fall upon his eyes, except for the momentary examination of his physicians, and it was with difficulty that Dr. Flint and myself obtained even a momentary glance at the eyes. The patient sedulously shut out light; his windows were not only darkened by the shutters, but dark curtains were hung across every avenue by which light might reach the room. The fire was perfectly screened. In addition to these measures, the patient wore dark goggles, with several folds of black silk interposed between the eyes and the glasses. From these extraordinary measures, the reader may infer the extreme extent of photophobia in this case. It was infinitely beyond any thing I had ever known then, or any thiug I have seen since. Another untoward symptom in this case was the inability in the patient to sleep. For three years he had never slept more than half an hour, in the twenty-four hours. In other respects the constitution, and the natural functions, seemed to be in good condition.

In consultation with Dr. Flint, it was determined to use Arnott's Hydrostatic bed, and to rely upon it mainly for the management of this singular case. To our great gratification, the patient slept soundly through seven consecutive hours the first night he was placed on the water-bed, and he commenced improving rapidly. He felt such great confidence in the powers of this new bed, that he soon followed the example of the recipients of miraculous favor, recorded in the histories of the Evangelists—he took up his bed and went home. It was a cumbrous affair, and the residence of the patient was a considerable distance from navigation. But Mr. D. had felt so much relief from the sleep secured by this water-bed, that he underwent all the difficulties, in transporting it over land, in order to be certain of a resource against suffering.

I have seen so much benefit from the water-bed, in other cases, besides the one detailed above, that I could not do less than commend it to the attention of practitioners. The lady of a distinguished clergyman in this city, after months of extreme affliction, had her bed of suffering made easy, and her dying agonies soothed by the water-bed—the Rev. Benj. O. Peers, in his dying hours, found a comfort in Dr. Arnott's Hydrostatic bed,* that had been sought in vain, from other sources.

In some recent numbers of the London Lancet, there are several suggestions connected with beds for the afflicted, that are worthy of notice. J. Henry Rogers, Esq., surgeon, thus speaks of what is called Cottam's spring-bed:

"In the autumn of 1846, during the time I was acting as house-surgeon of the Middlesex Hospital, some beds were sent there for trial by the inventor, which had been constructed by him with the view of securing all the advantages of the Arnot bed, without being liable to similar objections. These beds consisted of a light iron frame,

---

*A description of this bed may be found in the 2d volume of Arnott's "Elements of Physics."

with transverse elastic steel bars, to which were attached a number of iron springs placed vertically, supporting a thin detached horse-hair mattrass. As these springs act independently of each other, and yield in proportion to the pressure from above, the mattrass adapts itself perfectly to the surface of the body, the pressure being as equally diffused as in the water-bed. As the body of the patient is separated from the air merely by a thin mattrass, evaporation goes on as freely below as above, so that no accumulation of moisture can possibly take place —an advantage which was speedily discovered by the first patient who was placed on one of these beds, and who had been for some time confined to an Arnott bed. These beds being found fully to answer the expectations formed of them, have since been generally employed in the hospital in all cases where the Arnott bed was formerly had recourse to. There are now sixteen in constant use in that establishment; they are portable, cheap, and not liable to get out of order. I saw one during a recent visit to London, which had been in constant use since the autumn of 1846, and was in as serviceable a condition as when first sent in."

But the most useful article of this kind, is a water-cushion, invented by a Mr. Hooper. It is of an oblong form, and about 20 inches by sixteen in size. It is made of vulcanized India rubber, and is as soft as velvet. The space between the under and upper surface of the cushion should be about one inch, or one inch and a half. The cushion should be provided with a tube, with a stop-cock and funnel, by which the cushion may be filled or emptied, or the temperature of the water may be changed, without incommoding the patient.

There are many cases in which these water cushions are invaluable. Mr. Sampson says, "the water cushion is valuable in fever as a refreshing pillow for the head, in acute rheumatism as a cool support for the inflamed limb, as also in certain uterine affections, inflamed piles, &c., and when filled with hot water, it furnishes a most convenient means of imparting warmth to the spine."

Mr. Ceasar Hawkins, of St. George's Hospital, urges the use of the water cushion, "where the sacrum and hips are threatenened with sloughing, or in which sloughing has taken place; it is employed where the scapulæ, or spine, or heels are in danger, and where the os coccygis and ischia require defence. In injuries, such as fractures, or diseased joints, the arm or the leg may be laid lengthwise, with advantage on the water-pillow, to protect the elbow or heel from the effects of pressure.

The reader cannot fail to estimate properly the important character of the water-cushion. It possesses means of comfort for patients, that may be sought in vain without it.

*Intestinal obstruction, arising from disease of the sigmoid flexure of the colon and the rectum, in which the descending colon was successfully opened in the loin.*—Two interesting cases of this kind, are detailed, in the April number of the London Lancet, by T. Field, and J. Clarkson, Esqs., Members of the Royal College of Surgeons. The first case occurred in the person of a corpulent, muscular forger of coach axles. He was thirty-three years of age. He had always enjoyed good health, until within a year past, about the beginning of which time he began to suffer from pain in the bowels, constipation, and tenesmus, his stools becoming scanty, and voided with difficulty." For months these symptoms continued, becoming more aggravated in form. The patient often vomited after eating. Four days before Mr. Field's visit, the bowels had ceased to act, and all the symptoms became greatly aggravated. The abdomen was distended and tympanitic; there were pain, tenderness and some bulging over the transverse colon; the pain was paroxysmal, and accompanied by strong tenesmus; vomiting was almost incessant. Various and appropriate means were resorted to for opening the bowels, and these measures were continued. unsuccessfully, for twelve days. The case then assumed alarming symptoms, and Mr. Field determined upon an operation. "The patient was extended on a bed, with his face downwards, and a transverse incision was made in the left loin, commencing about two inches from the spine, and carried directly outwards for five inches and a half, about one finger's breadth above the crest of the ilium. The skin, fat, latissimus dorsi muscle and quadratus lumborum were successfully divided, and a shining membrane exposed." The bowel was found, at considerable depth; sutures were passed through it, to retain it in its position, and subsequently to affix it to the edges of the wound, and incision was then made into it, to the extent of half an inch, which immediately gave exit to an immense quantity of light-colored fluid feces. The patient was at once relieved from all distressing symptoms. The opening in the bowel was fixed with sutures to the skin, a large bread poultice was placed over the wound, and the patient enjoined to lie on his left side. Through the following night the evacuations continued abundant; the belly became soft and free from tenderness, and the general symptoms were still farther relieved. On the third day after the operation, the wound was rather inactive; it was syringed with yeast mixed with warm water, and dressed with lint and oil. Granulations sprung up, and the patient recovered, and resumed his trade, which required great physical exertion. He was occasionally constipated, caused, apparently, by a contraction of the artificial anus, which was relieved by injections of warm water.

This patient lived 21 months after this operation. The *post mortem* revealed a stricture, about 4 inches long, in the sigmoid flexure of the colon, this portion being filled with coagulable lymph.

The other case occurred in a robust, healthy looking woman, aged twenty-one. It is unnecessary to enter into the details of this case, as those, reported for Mr. Field, are very copious, and clearly mark the justifying symptoms for the operation, and the character of the operation. Mr. F.'s patient recovered her health for a time, and survived the operation fourteen months. In this case: "the obstruction was about six inches from the anus, and on a level with the fundus of the uterus. It consisted of dense, cartilaginous substance, surrounding the intestine, and completely obliterated the canal to the extent of half an inch." There was a great deal of diseased appearances in various parts of the abdominal viscera.

In the debates of the Royal Medical and Chirurgical Society, drawn forth by these cases, various members urged the importance of an early operation in such cases. Drastic purgatives, for obstructed bowels, are prejudicial, and much judgement is required, in order to determine how long purgative measures shall be persevered in, and when the necessity for the operation is absolute. There must enter into the material for making a judgment, the fact, that malignant disease is often connected with these obstructions. The whole subject is one of great interest to the physician, for lives are often lost in these cases, that should be saved.

Mr. Hodgson said, during the debate, that vomiting does not occur as early in the disease, when the obstruction is in the large intestines, as when the small intestines were the seat of it. The entire matter involved in these cases is worthy of the study of the physician, and it is respectfully commended to the readers of this Journal.

